# Effects of Anti-Calcitonin Gene-Related Peptide for Migraines: A Systematic Review with Meta-Analysis of Randomized Clinical Trials

**DOI:** 10.3390/ijms20143527

**Published:** 2019-07-18

**Authors:** I-Hsin Huang, Po-Chien Wu, En-Yuan Lin, Chien-Yu Chen, Yi-No Kang

**Affiliations:** 1 School of Medicine, College of Medicine, Taipei Medical University, Taipei 110, Taiwan; 2 Department of Education, Taipei Medical University Hospital, Taipei 110, Taiwan; 3 Division of Neurosurgery, Department of Surgery, Taiwan Adventist Hospital, Taipei 105, Taiwan; 4 Department of Anesthesiology, School of Medicine, College of Medicine, Taipei Medical University, Taipei 110, Taiwan; 5 Department of Anesthesiology, Taipei Medical University Hospital, Taipei 110, Taiwan; 6 Evidence-Based Medicine Center, Wan Fang Hospital, Taipei Medical University, Taipei 116, Taiwan

**Keywords:** migraine, anti-calcitonin gene-related peptide, internal medicine

## Abstract

We aimed to evaluate the response rate of migraines by using anti-calcitonin gene-related peptide (anti-CGRP) for patients with migraines. We searched three main medical databases up to 29 March 2019. No restriction on language and publication time were applied. Eligible trials included randomized clinical trials investigating a 50%, 75%, and 100% response rate of migraine patients after anti-CGRP intervention. The collected data were dichotomous, and risk ratios (RRs) with a 95% confidence interval (CI) were used to present the quantitative synthesis results. The systematic review identified 16 eligible randomized clinical trials (RCTs) with 9439 patients. Eight of the 16 trials with 2516 patients reported a 50% response rate, and the pooled results showed a significant benefit from anti-CGRP. However, the effects seem to gradually reduce from the first month (RR 1.99, 95% CI 1.59 to 2.49) to the third month (RR 1.48, 95% CI 1.26 to 1.75) of treatment. The magnitude of effect was influenced by the type of anti-CGRP, according to the test for differences between subgroups (I-square = 53%). The funnel plots and Egger’s tests did not show serious small study effects in the results. In conclusion, the current evidences confirmed that anti-CGRP treatment can reduce migraine pain in the short term (within three months), but the long-term effect should be investigated in the future. Moreover, its effects may be influenced by the type and dose of anti-CGRP. Therefore, future studies should make direct comparisons among anti-CGRP medications.

## 1. Introduction

Migraine is a highly prevalent, disabling neurological disorder with many socioeconomic and personal impacts [1]. It is characterized by headache, which is often accompanied with nausea, vomiting, photophobia, or prodromal and postdromal symptoms [1,2,3]. Migraine has become a main leading cause of disability [4]. Besides traditional acute migraine medications, anti-calcitonin gene-related peptide (anti-CGRP) medication has recently been developing as a new emerging treatment.

There are mainly two theories to explain the mechanism of migraine: The central neuronal theory and the vascular theory [5]. CGRP, a 37-amino acid neuropeptide, plays an important role in migraine pathophysiology. CGRP is a highly potent vasodilator in the trigeminovascular system, exacerbating vasodilation while causing neurogenic inflammation [5,6]. Nowadays, preventive treatment of migraine is mostly non-specific migraine medications and has had substantial adverse events, which may increase the non-adherence rate and controversial efficacy of the treatment [7]. Therefore, a migraine-specific, effective, well-tolerated medication is needed as a better preventive treatment.

Accordingly, human monoclonal antibodies acting as CGRP antagonists, such as Erenumab (AMG334), Eptinezumab (ALD403), Galcanezumab (LY2951742), and Fremanezumab (TEV48125), were developed to be an option of acute migraine treatment [8]. Several randomized clinical trials assessed the efficacy and safety of CGRP antagonists for chronic or episodic migraine. In the past two years, those trials reported their results at many conferences [9,10,11,12,13,14,15,16,17,18,19,20,21,22,23,24,25,26,27,28,29,30,31,32,33,34,35,36,37,38], and the full reports were recently published. Primary endpoints in these randomized clinical trials usually included a response rate of migraine days, monthly migraine day, and monthly headache day. Secondary endpoints included the usage times of migraine-specific medication, cumulative hours of headache, Headache Impact Test 6 (HIT-6) score, and Migraine Specific Quality of Life Questionnaire (MSQ) score. All the trials indicated better outcomes and prevention of migraine with anti-CGRP medication, but the efficacy seemed to be diversified by the different interval and period of drug administration [39,40,41,42,43,44,45,46,47,48,49,50,51,52,53,54,55,56,57,58,59,60,61,62,63,64]. Though the synthesized evidences consistently confirmed that anti-CGRP had no serious adverse events reported and the effects of anti-CGRP remain controversial due to differences in follow-up and the percentage of response rate [65,66,67,68,69]. Some previous meta-analyses and systematic reviews analyzed a small amount of the trials [67,68], and one article containing a larger amount of trials showed results with large heterogeneity by estimated analysis due to the variety of features and units of different trials [65]. A bigger synthesis pooling of more than 2500 cases showed that anti-CGRP had significantly better outcomes than placebo in the change of migraine days from baseline to the third month, but the insufficient evidence on the 100% responder rate was not statistically significant [66]. Additionally, there are several ongoing trials that should be updated. Consequently, our systematic review and meta-analysis aims to update the evidence of the effects of anti-CGRP for migraine by synthesizing the current randomized clinical trials.

## 2. Results

**Figure 1:**
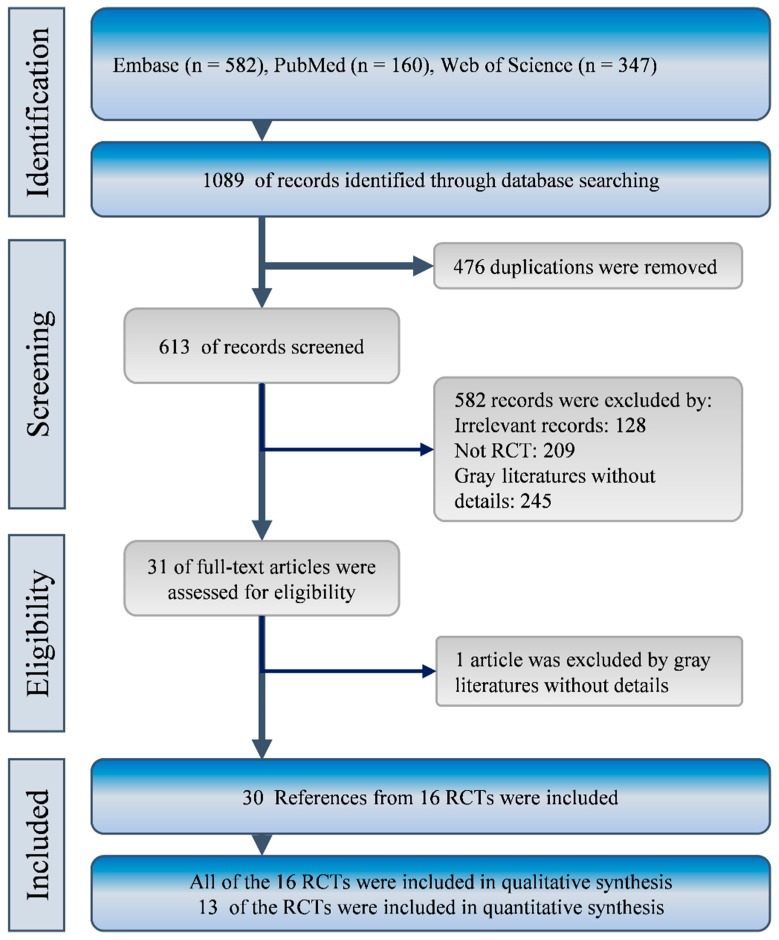
Flow diagram of study selection.

A total of 1089 studies were identified from the three important biomedical databases, in which 476 were duplicated. In the remaining 613 studies, 582 were removed after title, abstract, and article type screening because of irrelevance (*n* = 128), non-RCT study (*n* = 209), or gray literatures without details (*n* = 245). Then, we retrieved the full-text of the 31 remaining studies for further review. One study met the exclusion criteria and was removed [9]. Finally, the eligible studies were checked for data sources and they were found to be from 16 RCTs. These trials were included in this study for qualitative and quantitative synthesis [39,40,41,42,43,44,45,46,47,48,49,50,52,53,54,55,56,57,58,59,60,61,62,63,64]. The flow diagram of evidence selection is presented in Figure 1.

### 2.1. Characteristics and Quality of Included Studies

The 16 included RCTs recruited 9439 patients with migraine from Argentina, Canada, Europe, Israel, Korea, Mexico, Russia, Taiwan, Turkey, and the USA between July 2012 and October 2017. Table 1 presents characteristics of each trial. These trials gave anti-CGRP for at least 12 weeks, and the longest treatment duration was 52 weeks. The trials completed a follow-up of at least four weeks, and the longest follow-up duration was four months. Eleven trials focused on episodic migraine, and four trials investigated chronic migraine. The other one recruited both populations of episodic migraine and chronic migraine. These trials did not set criteria for aura (Table S1). The age of patients ranged from 18 to 70 years old. Most of the patients were females (*n* = 7992; 84.67%), and there were only 1447 males (15.33%). Most trials in this systematic review and meta-analysis presented a low selection bias, performance bias, attrition bias, and reporting bias (Table S2).

### 2.2. Monthly 50% Response Rate

**Figure 2:**
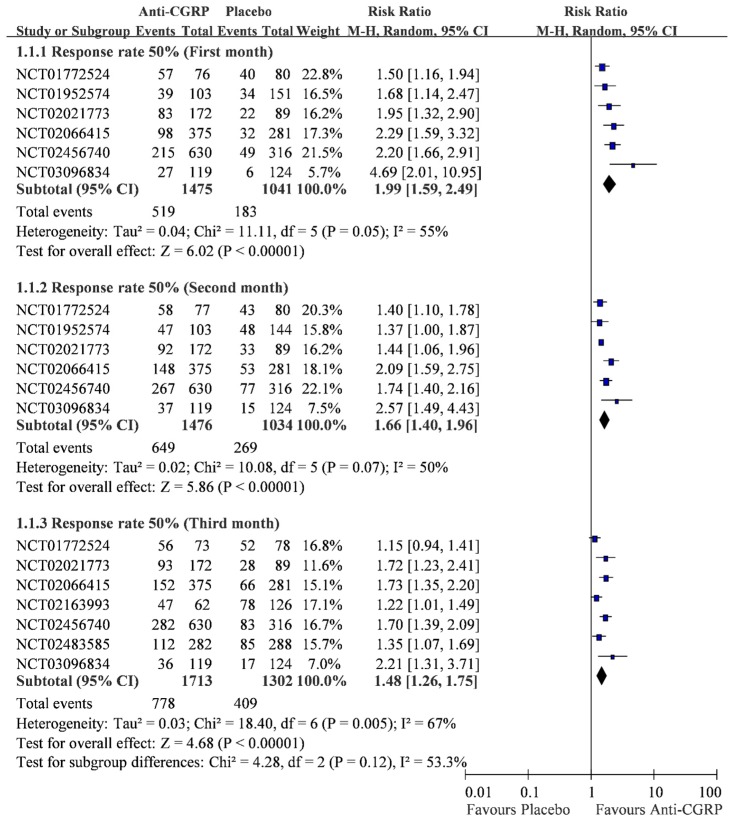
The 50% reduction rate of anti-CGRP and placebo.

Out of the 16 RCTs, eight trials reported data of the monthly 50% response rate (Figure 2) [39,40,41,42,43,44,45,48,50,53,57,58,64]. According to the data from 2516 patients, anti-CGRP led to a significantly higher 50% response rate when compared with placebo (RR 1.99, 95% CI 1.59 to 2.49) in the first month. In the second month, anti-CGRP also resulted in a significantly higher 50% response rate (649/1476; 43.97%) than placebo (269/1034; 26.02%) with RR 1.66 (95% CI 1.40 to 1.96). Similarly, anti-CGRP showed a significantly higher 50% response rate (778/1713; 45.42%) than placebo (409/1302; 31.41%) with RR 1.48 (95% CI 1.26 to 1.75) in the third month. Although the anti-CGRP showed a better 50% response rate without small study effects (Table 2 and Figure S1–S3), a slightly decreasing trend could be observed from the first month (RR 1.99), to the second month (RR 1.66) and the third month (RR 1.48). The test for subgroup differences also reflected a high heterogeneity among the three subsets (I-square = 53.3%). Furthermore, the heterogeneity in these outcomes reached the threshold (I-square = 50%), and the effect of heterogeneity should be concerning. Thus, subset analysis was applied to explore the source of heterogeneity.

The subset analysis of drugs decreased heterogeneity from I-square 55% to 40.42% in the first month, from 50% to 49.38% in the second month, and from 67% to 31.79% in the third month (Table 2 and Figure S4–S6). The results of the subset analysis still confirmed the effect of anti-CGRP medications on migraine, except for Eptinezumab in the third month. The effect of Eptinezumab on migraine in the third month should be further tested in the future because the only available RCT for our review consisted of a small sample size (*n* = 151).

### 2.3. Cumulative Response Rate within Three Months

**Figure 3:**
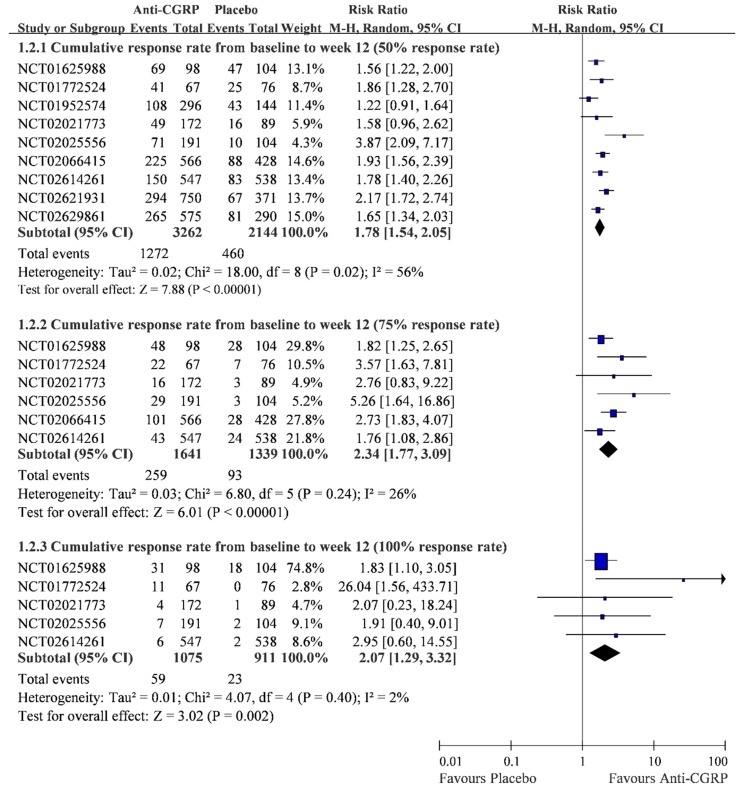
Cumulative response rate from the initial to the 12th month between anti-CGRP and placebo.

A total of nine RCTs presented a cumulative response rate within three months [39,41,42,43,44,46,47,48,49,50,55,57,59,61,62]. These trials recruited 5406 cases, and the pooled results are shown in Figure 3. The anti-CGRP (1272/3262; 38.99%) had a significantly higher rate in a 50% cumulative reduction of migraine within three months when it was compared with placebo (460/2144; 21.46%) (RR 1.78, 95% CI 1.54 to 2.05). Similarly, the pooled result indicated that anti-CGRP (259/1641; 15.78%) had significantly higher rates in the 75% response within three months than placebo (93/1339; 6.95%) (RR 2.34, 95% CI 1.77 to 3.09). For the 100% response rate within three months, the pooled result also showed that anti-CGRP (59/1075; 5.49%) was significantly higher than placebo (23/911; 2.52%) (RR 2.07, 95% CI 1.29 to 3.32). Egger’s test did not reflect a small study effect on these results (Table 2 and Figure S7–S9). Low heterogeneities were detected in the results of the 75% response rate (I-square = 26%) and 100% response rate (I-square = 2%), but the 50% response rate still had a high heterogeneity (I-square = 56%; *p* < 0.10). Although this study tried to reduce the heterogeneity by stratifying the anti-CGRP medications, the heterogeneity was not successfully reduced (Table 2). Unfortunately, the heterogeneity in the subset of Erenumab (I-square = 83.97%; *p* < 0.10) and Frenamezumab (I-square = 66.02%; *p* < 0.10) were still high (Table 2 and Figure S10–S12).

## 3. Discussion

In this study, we synthesized 16 trials. Our data showed that, as compared with placebo, treatment with anti-CGRP medications was associated with a significant progressive decrease of the response rate of migraine days during the three-month period. Though the heterogeneity is low in the overall three-month analysis data, the I-square is quite high (51.4%), reflecting the differences between months and types of anti-CGRP medications. According to the Figure 2, the efficacy of medications decreased through time, showing a slightly descending trend. Moreover, there was an individual difference in each four types of the anti-CGRP medications. Among them, Frenamezumab had the least efficacy. In other words, anti-CGRP medications showed effective results in treating migraine, but the efficacy may be dependent on the time and types of medications used.

The neuropeptide calcitonin gene-related peptide acts as a significant biomarker in the trigeminovascular system. Initially, an oral CGRP inhibitor, geptan, was developed to prevent migraine, but the trials of geptan were terminated due to liver toxicity. Another anti-CGRP medication, fully human monoclonal antibody, was developed alternatively [8]. Three monoclonal antibodies, including galcanezumab, fremanezumab, and eptinezumab, bind to become an isoform of CGRP, preventing CGRP from binding to its receptor and activating the pathway of vasodilation and inflammation [47,70]. The other monoclonal antibody, erenumab, binds to CGRP receptors directly and can also prevent the activation of trigeminal fibers [40,70]. Since the pharmacological properties of these monoclonal antibodies are different, they lead to a various efficacy of migraine control and prevention. The characteristics among these anti-CGRP can vary from target selection (CGRP or CGRP receptor), mechanism of immune activation, pharmacokinetics, and bioavailability.

Among three antibodies that bind to become an isoform of CGRP, Eptinezumab, Galcanezumab, and Fremanezumab are Immunoglobulin G1 (IgG1)antibody, IgG4 antibody, and IgG2 antibody, respectively [39,47,70,71]. Erenumab is an IgG2 antibody and competitively binds to CGRP receptors [40,70]. Additionally, only Eptinezumab is given in intravenous (IV) form, while the others are subcutaneous injection [72]. According to our results, subcutaneous injection of IgG2 antibody, Fremanezumab, performed with the least efficacy among four anti-CGRP medications. Another possible reason to explain the variety of efficacy is that Fremanezumab has the longest half-life among the four antibodies, ranging from 31 to 39 days [70,73,74]. However, further studies are warranted to evaluate the pharmacokinetic comparison of anti-CGRP medication to explain why Fremanezumab had less efficacy than the other monoclonal antibodies.

Anti-CGRP medications have a unique mechanism toward the suppression of migraine pain in comparison to traditional migraine therapy. Anti-CGRP medications, as fully human monoclonal antibodies, selectively bind to CGRP receptors to prevent activation of trigeminal fibers from vasodilation and inflammation. On the other hand, traditional migraine medication therapy, including triptans, ergotamines, acetaminophen, and non-steroidal anti-inflammatory drugs, aims to block 5HT-1B and 5HT-1D receptors or prevent inflammatory substances from producing via the COX pathway, which often lead to adverse effects due to systemic action of these pathways. Current studies have shown that traditional migraine therapy might correlate with a deterioration of the patient’s symptoms. Though it has not been fully proved yet, anti-CGRP medications are an expecting and rising therapy in reducing the number of pain days, promoting quality of life, and even preventing pain from happening in patients with migraine.

### Comparison to the Previous Syntheses

The previous five syntheses on this topic concluded that anti-CGRP is effective and safe, yet these evidences did not completely mention the effects in each month [65,66,67,68,69]. Three of these syntheses involved cases of more than 2500 [65,66,69], and the other two pooled limited data that amounted to fewer than 1000 cases [67,68]. One of the three syntheses with a bigger sample size did not conduct quantitative synthesis [69]. The other two of the three syntheses provided stronger evidence on the effects of anti-CGRP for migraine by identifying more evidences on this topic. Interestingly, one of the two meta-analyses included eight trials in April 2018, and the last updated meta-analysis only included five trials using similar criteria in July 2018. Although the two meta-analyses made similar conclusions, the meta-analysis with the bigger sample size reported heterogeneous results. The sources of the heterogeneities should be explored, yet the previous analysis did not successfully identify the sources of the heterogeneities.

The present meta-analysis improved evidence on this topic by identifying 16 trials with 9439 patients with migraine, synthesizing more cases, and pointing out the potential sources of heterogeneities. The pooled results on the response rate of migraine were based on at least 2500 cases, and the result of the 50% cumulative response rate was based on 5406 cases. This pooling gave a clear picture of the efficacy of anti-CGRP on migraine, and no evidence detected small study effects on the results. Moreover, the present quantitative syntheses for the response rate were successfully reduced heterogeneities (I-square < 50%). That is to say, the present meta-analysis is more confident and informative than previous syntheses.

## 4. Methods

This is a prospective systematic review starting from 28 November 2018, and study protocol was written beforehand. The primary design was registered on PROSPERO (CRD42018118063). The research team involved a neurologist, an anesthesiologist, and an experienced researcher in systematic review and meta-analysis [75,76,77]. The neurologist and anesthesiologist also have experience on conducting systematic reviews and meta-analyses [78,79]. The authors conducted this study according to the PRISMA guidelines in evidence selection, quality assessment, evidence synthesis, and research reporting [80].

### 4.1. Data Source and Search

Basic eligible criteria for evidence selection was defined before a comprehensive search was conducted. The inclusion criteria were as follows: (1) Studies recruiting patients with migraine, and (2) studies evaluating the outcomes of patients who received treatment with anti-CGRP medications. According to these criteria, the relevant terms of migraine, Eptinezumab, Erenumab, Galcanezumab, and Fremanezumab, in free-text, medical subject headings (MeSH in PubMed and Emtree in EMBASE), and abbreviations were used for the literature search. The keywords were combined by appropriate Boolean operators, and formed a primary search strategy without limitations on the language and publish data. The primary search strategy was done with PubMed, and was adapted to the Cochrane Library (including Cochrane CENTERL), Embase, and Web of Science. The final search was completed on 29 March 2019 (Table S3).

### 4.2. Study Selection

After potential studies were identified, two authors (YHH and BCW) rooted out irrelevant studies by screening the title and abstract according to the exclusion criteria. The exclusion criteria included: (1) Studies recruiting patients with diseases other than migraine, (2) studies mixing patients with general headache and migraine without stratification analysis, (3) studies using treatments other than anti-CGRP medication, (4) gray literature studies without details of patients’ characteristics or results, and (5) studies that were not RCTs. The corresponding author (YNK) made the final judgement for study selection when the two authors had any disagreement.

### 4.3. Data Extraction and Quality Assessment

The two authors (YHH and BCW) also individually reviewed all selected RCTs for data extraction and risk of bias assessment. They extracted trial characteristics and outcome data. The characteristics involved trial registry number, country, recruitment duration, medication strategy, age range, and sex. The outcome data involved the 50%, 75%, and 100% response rate of migraine. The risk of bias of the selected RCTs were assessed by using the Cochrane Risk of Bias Tool. This tool comprises seven methodological items, including: (1) Allocation generation, (2) allocation concealment, (3) blinding of participants and personnel, (4) blinding of outcome assessment, (5) incomplete outcome data, (6) selective report, and (7) other bias. The third author (YNK) made the final judgement for the risk of biased assessment.

### 4.4. Data Synthesis and Analysis

The quantitative synthesis in this study used the risk ratio (RR) for performing binary outcome analysis from the RCTs. This study chose the Mantel–Haenszel method for meta-analysis. Generally, the Mantel–Haenszel method is considered to be preferable to the inverse variance method. All the analyses were conducted using the random-effects model. The results are shown in the RR and 95% confidence interval (CI).

To assess the quality of the pooling results, this study detected the heterogeneity and small study effect. I-square and the *p*-value of Cochran Q was used to assess heterogeneity. This study defined high heterogeneity as an I-square higher than 50% or the *p*-value of Cochran Q lower than 0.10 (a rigorous threshold for heterogeneity detection). To explore the source of heterogeneity, this quantitative synthesis conducted sensitivity analysis by using the subset design. The subset was stratified according to different types of drugs. A small study effect was illustrated using the funnel plot and calculated Egger’s test. Pooled results were deemed affected by small study bias when the *p*-value of Egger’s test lower than 0.05.

## 5. Limitations

Although this systematic review and meta-analysis owned more advantages than previous syntheses, this study has three limitations. First, this meta-analysis cannot distinguish the effects from different dosages because the dosages among different types of anti-CGRP treatments cannot be converted easily. Dosage effects was also a limitation in the previous syntheses. Therefore, further studies should investigate dosage effects among different type of anti-CGRP treatments. Secondly, this meta-analysis did not synthesize the monthly migraine days, reduction of migraine days, monthly headache days, or reduction of headache days. This limitation may result in a lack of intuitive information (mean difference), but using the response rate can keep results unaffected by an extreme value. Moreover, response rates presenting the percentage of reduction in migraine days could be an index of the improvement. Thirdly, few evidences reported a 75% or 100% response rate each month. Thus, this meta-analysis cannot give a clear picture about how the anti-CGRP reaches a 75% or 100% response rate of migraine monthly. However, this study still proved an overview showing that the anti-CGRP is a highly effective treatment for migraine according to the cumulative 75% and 100% response rate.

## 6. Conclusions

In conclusion, the current evidences confirmed that anti-CGRP reduced migraine in the short term (within three months). The long-term effect should be investigated in the future. Moreover, its effects may be affected by the type and use of anti-CGRP. Therefore, future studies should make direct comparisons among the anti-CGRP medications.

## Figures and Tables

**Table 1:** Characteristics of the included randomized controlled trials.

Trial	Area	Recruitment Duration	Medication	Patients (*n*)	Age	Male/Female
NCT01772524 [39]	USA	Jan. 28, 2013 ~ Dec. 23, 2013	Eptinezumab 1000 mg/placebo	163	18–55	30/133
NCT02456740 [40,57]	Canada, Europe, Turkey, USA	Jul. 2015 ~ Sep. 5, 2016	Erenumab 70 mg/140 mg/placebo	955	18–65	141/814
NCT01952574 [41,44]	Canada, Europe, USA	Aug. 6, 2013 ~ June 30, 2014	Erenumab 7 mg/21 mg/70 mg/placebo	483	18–60	94/389
NCT02066415 [42,43,57]	Canada, Europe, USA	Apr. 3, 2014 ~ Dec. 4, 2015	Erenumab 70 mg/140 mg/placebo	667	18–65	115/552
NCT02483585 [45]	Canada, Europe, USA	Jul. 2015 ~ Jul. 2016	Erenumab 70 mg/placebo	577	18–65	85/492
NCT03096834 [58]	Australia, Europe	Mar. 20, 2017 ~ Oct. 27, 2017	Erenumab 140 mg/placebo	246	18–65	46/200
NCT02629861 [46]	Canada, Europe, Russia, USA	Mar. 23, 2016 ~ Apr. 10, 2017	Fremanezumab 225 mg monthly/ 3·225 mg single higher dose/placebo	875	18–70	133/742
NCT02621931 [47]	USA	Mar. 2016 ~ Jan. 2017	Fremanezumab 675 mg + 2·225 mg/ 675 mg + 2·placebo/placebo	1130	18–70	139/991
NCT02021773 [48,49,50,51]	USA	Jan. 2014 ~ Dec. 2014	Fremanezumab 900 mg/675-225 mg/placebo	263	18–65	37/226
NCT02025556 [59]	USA	Jan. 2014 ~ Jan. 2015	Fremanezumab 675 mg/225 mg/placebo	297	18–65	36/261
NCT02614183 [52,60,61]	Canada, USA	Jan. 11, 2016 ~ Mar. 22, 2017	Galcanezumab 120 mg/240 mg/placebo	858	18–65	140/818
NCT02163993 [53,56,64]	USA	July 7, 2014 ~ Aug. 19, 2015	Galcanezumab 5 mg/50 mg/120 mg/ 300 mg/placebo	410	18–65	70/340
NCT01625988 [55]	USA	July 31, 2012 ~ Sep. 18, 2013	Galcanezumab 150 mg/placebo	217	18–65	33/184
NCT02614196 [54,60,61]	Argentina, Europe, Israel, Korea, Mexico, Taiwan, USA	Jan. 2016 ~ Mar. 2017	Galcanezumab 120 mg/240 mg/placebo	915	18–65	134/781
NCT02614261 [61,62]	Argentina, Canada, Europe, Israel, Mexico, Taiwan, USA	Jan. 2016 ~ Mar. 2017	Galcanezumab 120 mg/240 mg/placebo	1113	18–65	167/946
NCT02614287 [63]	Canada, Europe, USA	Dec. 2015 ~ Sep. 2017	Galcanezumab 120 mg/240 mg/placebo	270	18–65	47/223

**Table 2:** Summary of further analysis.

Outcome			Effect	95% CI ^2^		Heterogeneity		Small study bias	
(Subset)	Study	Patients	Size ^1^	Lower	Upper	I ^2^ (%)	*p*	*Estimate*	*p*
Response rate 50% (First month)								3.014	0.119
Eptinezumab	1	156	1.5	1.163	1.935	0	NE		
Erenumab	4	2099	2.206	1.699	2.866	40.42	0.169		
Frenamezumab	1	261	1.952	1.316	2.895	0	NE		
Response rate 50% (Second month)								1.649	0.532
Eptinezumab	1	157	1.401	1.102	1.782	0	NE		
Erenumab	4	2092	1.81	1.459	2.246	49.38	0.115		
Frenamezumab	1	261	1.443	1.064	1.956	0	NE		
Response rate 50% (Third month)								3.531	0.147
Eptinezumab	1	151	1.151	0.941	1.408	0	NE		
Erenumab	4	2415	1.628	1.39	1.908	31.79	0.222		
Frenamezumab	1	261	1.719	1.228	2.405	0	NE		
Galcanezumab	1	188	1.225	1.006	1.49	0	NE		
Response rate 50% (From baseline to week 12)								1.089	0.544
Eptinezumab	1	143	1.86	1.281	2.703	0	NE		
Erenumab	2	1434	1.555	0.991	2.438	83.97	0.013		
Frenamezumab	4	2542	2.024	1.518	2.697	66.02	0.032		
Galcanezumab	2	1287	1.667	1.403	1.981	0	0.435		

^1^ Risk ratio. ^2^ CI, confidence interval; NE, not estimate.

## Supplementary Materials

Supplementary materials can be found at https://www.mdpi.com/1422-0067/20/14/3527/s1.

## Abbreviations

Anti-CGRP	anti-calcitonin gene-related peptide
CI	confidence interval
RCT	randomized clinical trial
RR	risk ratio
SAW	single active warming strategy
SUCRA	surface under the cumulative ranking curve
