# Supplementary material for: Effects of Anti-Calcitonin Gene-Related Peptide for Migraines: A Systematic Review with Meta-Analysis of Randomized Clinical Trials
Source: Int J Mol Sci. 2019 Jul 18;20(14):3527. doi: 10.3390/ijms20143527 (PMC6678090; doi:10.3390/ijms20143527)

Effects of anti-calcitonin gene related peptide on patients with migraine:

A systematic review with meta-analysis of randomized clinical trials

(Supplementary Materials)

Supplementary Material

Table S1. Further information of the included trials

Table S2. Risk of bias

Table S3. Search strategy

Figure S1. Small study effect test for 50% response rate (First month)

Figure S2. Small study effect test for 50% response rate (Second month)

Figure S3. Small study effect test for 50% response rate (Third month)

Figure S4. Subset analysis for 50% response rate (First month)

Figure S5. Subset analysis for 50% response rate (Second month)

Figure S6. Subset analysis for 50% response rate (Third month)

Figure S7. Small study effect test for Cumulative response rate (50%)

Figure S8. Small study effect test for Cumulative response rate (75%)

Figure S9. Small study effect test for Cumulative response rate (100%)

Figure S10. Subset analysis for cumulative response rate (50%)

Figure S11. Subset analysis for cumulative response rate (75%)

Figure S12. Subset analysis for cumulative response rate (100%)

Table S1
Further information of the included trials

Table S1
Further information of the included trials

Study	Episodic / Chronic	Aura	Intervention period	Follow-up
NCT01772524	Episodic	Without + with aura	12 weeks	12 weeks
NCT02456740	Episodic	Non-specific	28 weeks	12 weeks
NCT01952574	Episodic	Non-specific	12 weeks	12 weeks
NCT02066415	Chronic	Non-specific	12 weeks	12 weeks
NCT02483585	Episodic	Without + with aura	40 weeks	12 weeks
NCT03096834	Episodic	Without + with aura	12 weeks	12 weeks
NCT02629861	Episodic	Non-specific	12 weeks	12 weeks
NCT02621931	Chronic	Non-specific	12 weeks	12 weeks
NCT02021773	Chronic	Without + with aura	12 weeks	4 weeks
NCT02025556	Episodic	Without + with aura	12 weeks	4 weeks
NCT02614183	Episodic	Non-specific	26 weeks	18 weeks
NCT02163993	Episodic	Non-specific	12 weeks	12 weeks
NCT01625988	Episodic	Without + with aura	12 weeks	12 weeks
NCT02614196	Episodic	Non-specific	26 weeks	18 weeks
NCT02614261	Chronic	Without + with aura	52 weeks	18 weeks
NCT02614287	Episodic and chronic	Without + with aura	52 weeks	18 weeks

Table S2
Risk of bias

Table S2

Risk of bias summary

Study	1	2	3	4	5	6	7
NCT01772524	Low risk	Low risk	Low risk	Low risk	Low risk	Low risk	High risk
NCT02456740	Low risk	Low risk	Low risk	Low risk	Low risk	Low risk	High risk
NCT01952574	Low risk	Low risk	Low risk	High risk	Low risk	Low risk	High risk
NCT02066415	Low risk	Low risk	Low risk	High risk	Low risk	Low risk	High risk
NCT02483585	Low risk	Low risk	Low risk	Low risk	Low risk	Low risk	High risk
NCT03096834	Low risk	Low risk	Low risk	Low risk	Low risk	Low risk	High risk
NCT02629861	Low risk	Low risk	Low risk	Low risk	Low risk	Low risk	High risk
NCT02621931	Low risk	Low risk	Low risk	Low risk	Low risk	Low risk	High risk
NCT02021773	Low risk	Low risk	Low risk	Low risk	Low risk	Low risk	High risk
NCT02025556	Low risk	Low risk	Low risk	Low risk	Low risk	Low risk	High risk
NCT02614183	Low risk	Low risk	Low risk	Unclear	Low risk	Low risk	High risk
NCT02163993	Low risk	Unclear	Low risk	Low risk	Low risk	Low risk	High risk
NCT01625988	Low risk	Low risk	Low risk	Low risk	Low risk	Low risk	High risk
NCT02614196	Low risk	Low risk	Low risk	Low risk	Low risk	Low risk	High risk
NCT02614261	Low risk	Low risk	Unclear	Unclear	Low risk	Low risk	High risk
NCT02614287	Unclear	High risk	Unclear	Unclear	High risk	Low risk	High risk

1 sequence generation; 2 allocation concealment; 3 blinding of participants and personnel; 4 blinding of outcome assessment; 5 incomplete outcome data; 6 selective reporting; 7 other bias.

Table S3
Search strategy

Table S3
Search strategy

Primary search steps:

#1. migraine
#2. Erenumab OR OR OR OR OR ald403 OR tev 48125 OR tev48125 OR LY2951742 OR monoclonal anti-CGRP antibody OR monoclonal antibody to calcitonin gene-related peptide
#3. eptinezumab
#4. fremanezumab
#5. Galcanezumab
#6. ald 403
#7. ald403
#8. tev 48125
#9. tev48125
#10. LY2951742
#11. monoclonal anti-CGRP antibody
#12. monoclonal antibody to calcitonin gene-related peptide
#13. #2 OR #3 OR #4 OR #5 OR #6 OR #7 OR #8 OR #9 OR #10 OR #11 OR #12
#14. #1 AND #13

Final syntax in Embase:

('migraine'/exp OR migraine OR 'familial migraine' OR 'headache, migrainous' OR 'hemicrania' OR 'migraine' OR 'migraine disorders' OR 'status hemicranicus') AND ('erenumab'/exp OR erenumab OR 'amg 334' OR 'amg334' OR 'erenumab' OR 'monoclonal anti-cgrp antibody' OR 'monoclonal antibody to calcitonin gene-related peptide' OR 'eptinezumab'/exp OR eptinezumab OR 'ald 403' OR 'ald403' OR 'eptinezumab' OR 'fremanezumab'/exp OR fremanezumab OR 'fremanezumab' OR 'lbr 101' OR 'lbr101' OR 'rn 307' OR 'rn307' OR 'tev 48125' OR 'tev48125' OR 'galcanezumab'/exp OR 'galcanezumab' OR 'ly 2951742' OR 'ly2951742')

Final syntax in PubMed:

migraine AND (Erenumab OR eptinezumab OR fremanezumab OR Galcanezumab OR ald 403 OR ald403 OR tev 48125 OR tev48125 OR LY2951742 OR monoclonal anti-CGRP antibody OR monoclonal antibody to calcitonin gene-related peptide)

Final syntax in Web of Science:

Topic: migraine AND Topic: (Erenumab OR eptinezumab OR fremanezumab OR Galcanezumab OR ald 403 OR ald403 OR tev 48125 OR tev48125 OR LY2951742 OR monoclonal anti-CGRP antibody OR monoclonal antibody to calcitonin gene-related peptide)

Figure S1 to S3

Small study effect of the response rates

Figure S1

Small study effect test for 50% response rate (First month)

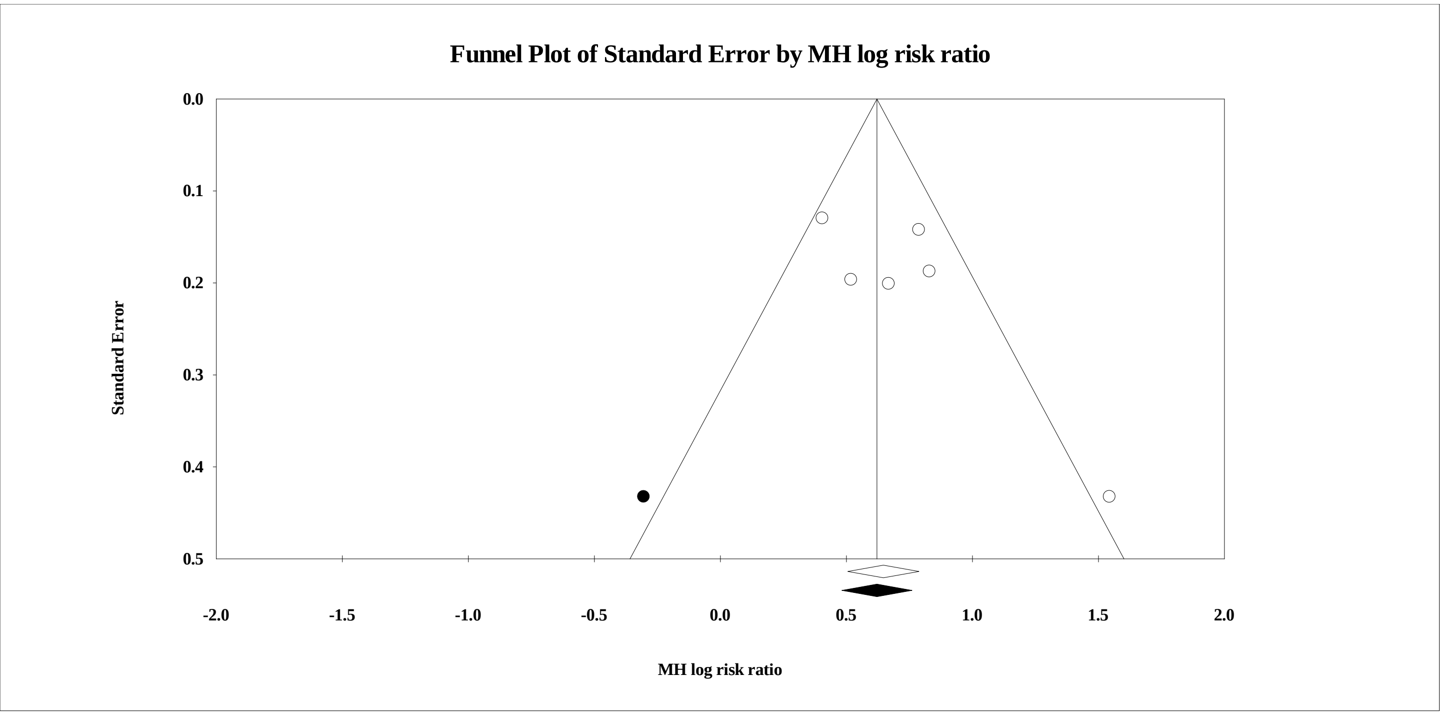

Egger's regression intercept

Intercept	3.01383
Standard error	1.52531
95% lower limit (2-tailed)	-1.22111
95% upper limit (2-tailed)	7.24878
t-value	1.97588
df	4.00000
P-value (1-tailed)	0.05968
P-value (2-tailed)	0.11936

Figure S2

Small study effect test for 50% response rate (Second month)

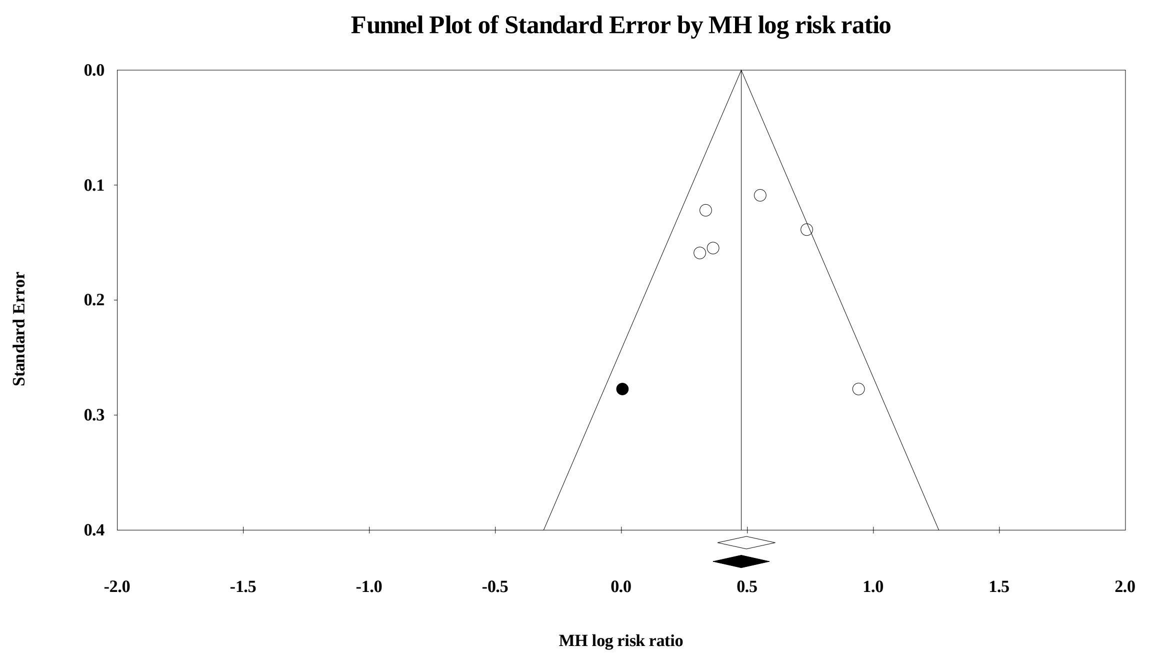

Egger's regression intercept

Intercept	1.64920
Standard error	2.41268
95% lower limit (2-tailed)	-5.04949
95% upper limit (2-tailed)	8.34788
t-value	0.68355
df	4.00000
P-value (1-tailed)	0.26590
P-value (2-tailed)	0.53180

Figure S3

Small study effect test for 50% response rate (Third month)

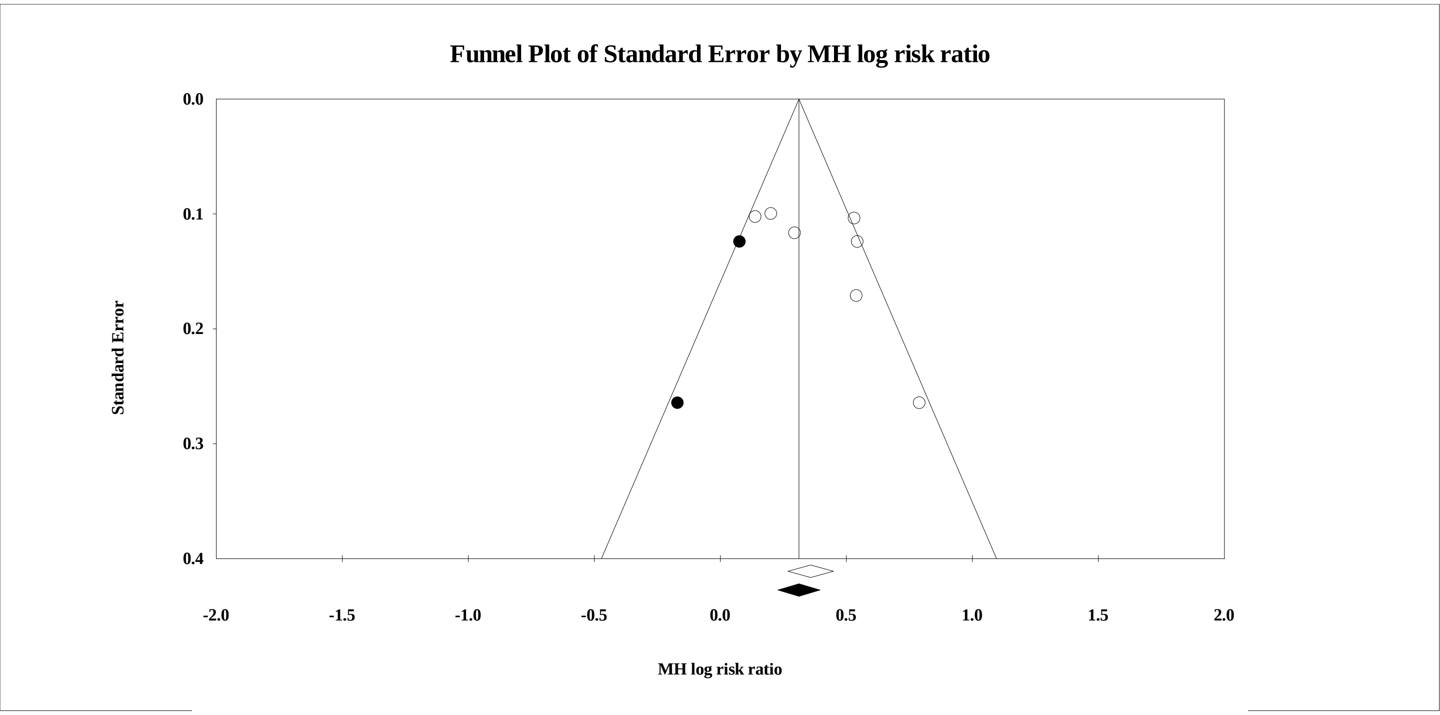

Egger's regression intercept

Intercept	3.53063
Standard error	2.05581
95% lower limit (2-tailed)	-1.75399
95% upper limit (2-tailed)	8.81524
t-value	1.71739
df	5.00000
P-value (1-tailed)	0.07328
P-value (2-tailed)	0.14655

Figure S4 to S6
Further analysis of 50% response rates

Figure S4
Subset analysis for 50% response rate (First month)

Subgroup	Anti-CGRP Events	Anti-CGRP Total	Placebo Events	Placebo Total	Weight	Risk Ratio M-H, Random, 95% CI
**2.1.1 Response rate 50%, week 4 (Eptinezumab)**						
NCT01772524	57	76	40	80	22.8%	1.50 [1.16, 1.94]
**Subtotal (95% CI)**		**76**		**80**	**22.8%**	**1.50 [1.16, 1.94]**
Total events	57		40			
Heterogeneity: Not applicable						
Test for overall effect: Z = 3.12 (P = 0.002)						
**2.1.2 Response rate 50%, week 4 (Erenumab)**						
NCT01952574	39	103	34	151	16.5%	1.68 [1.14, 2.47]
NCT02066415	98	375	32	281	17.3%	2.29 [1.59, 3.32]
NCT02456740	215	630	49	316	21.5%	2.20 [1.66, 2.91]
NCT03096834	27	119	6	124	5.7%	4.69 [2.01, 10.95]
**Subtotal (95% CI)**		**1227**		**872**	**61.0%**	**2.21 [1.70, 2.87]**
Total events	379		121			
Heterogeneity: Tau² = 0.03; Chi² = 5.04, df = 3 (P = 0.17); I² = 40%						
Test for overall effect: Z = 5.93 (P < 0.00001)						
**2.1.3 Response rate 50%, week 4 (Frenamezumab)**						
NCT02021773	83	172	22	89	16.2%	1.95 [1.32, 2.90]
**Subtotal (95% CI)**		**172**		**89**	**16.2%**	**1.95 [1.32, 2.90]**
Total events	83		22			
Heterogeneity: Not applicable						
Test for overall effect: Z = 3.33 (P = 0.0009)						
**Total (95% CI)**		**1475**		**1041**	**100.0%**	**1.99 [1.59, 2.49]**
Total events	519		183			
Heterogeneity: Tau² = 0.04; Chi² = 11.11, df = 5 (P = 0.05); I² = 55%						
Test for overall effect: Z = 6.02 (P < 0.00001)						
Test for subgroup differences: Chi² = 4.41, df = 2 (P = 0.11), I² = 54.6%						

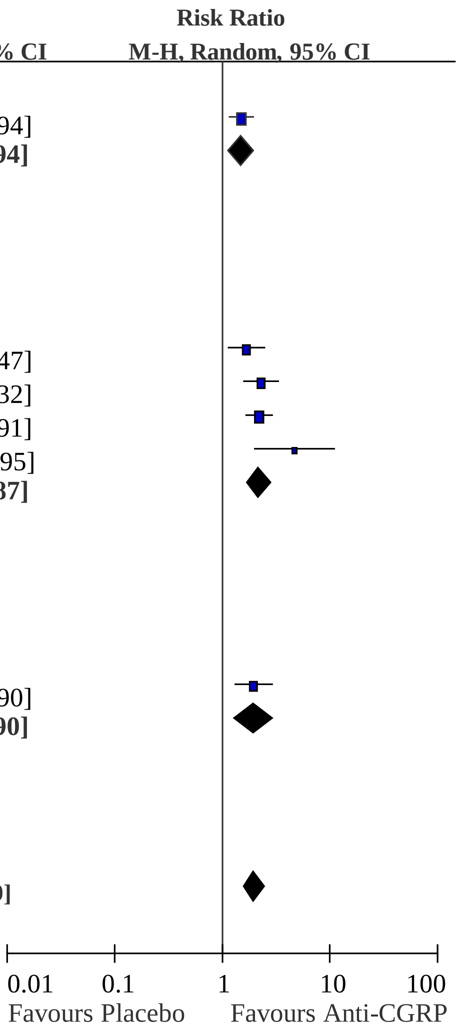

Figure S5

Subset analysis for 50% response rate (Second month)

Subgroup	Anti-CGRP Events	Anti-CGRP Total	Placebo Events	Placebo Total	Weight	Risk Ratio M-H, Random, 95% CI
**2.2.1 Response rate 50%, week 8 (Eptinezumab)**						
NCT01772524	58	77	43	80	20.3%	1.40 [1.10, 1.78]
**Subtotal (95% CI)**		**77**		**80**	**20.3%**	**1.40 [1.10, 1.78]**
Total events	58		43			
Heterogeneity: Not applicable						
Test for overall effect: Z = 2.75 (P = 0.006)						
**2.2.2 Response rate 50%, week 8 (Erenumab)**						
NCT01952574	47	103	48	144	15.8%	1.37 [1.00, 1.87]
NCT02066415	148	375	53	281	18.1%	2.09 [1.59, 2.75]
NCT02456740	267	630	77	316	22.1%	1.74 [1.40, 2.16]
NCT03096834	37	119	15	124	7.5%	2.57 [1.49, 4.43]
**Subtotal (95% CI)**		**1227**		**865**	**63.5%**	**1.81 [1.46, 2.25]**
Total events	499		193			
Heterogeneity: $Tau^2 = 0.02$; $Chi^2 = 5.93$, df = 3 (P = 0.12); $I^2 = 49\%$						
Test for overall effect: Z = 5.39 (P < 0.00001)						
**2.2.3 Response rate 50%, week 8 (Frenamezumab)**						
NCT02021773	92	172	33	89	16.2%	1.44 [1.06, 1.96]
**Subtotal (95% CI)**		**172**		**89**	**16.2%**	**1.44 [1.06, 1.96]**
Total events	92		33			
Heterogeneity: Not applicable						
Test for overall effect: Z = 2.36 (P = 0.02)						
**Total (95% CI)**		**1476**		**1034**	**100.0%**	**1.66 [1.40, 1.96]**
Total events	649		269			
Heterogeneity: $Tau^2 = 0.02$; $Chi^2 = 10.08$, df = 5 (P = 0.07); $I^2 = 50\%$						
Test for overall effect: Z = 5.86 (P < 0.00001)						
Test for subgroup differences: $Chi^2 = 2.83$, df = 2 (P = 0.24), $I^2 = 29.4\%$						

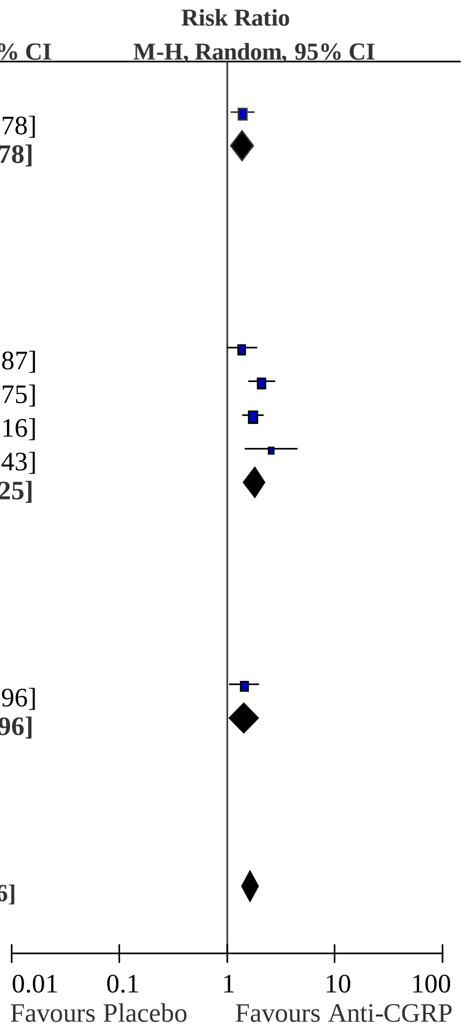

Figure S6

Subset analysis for 50% response rate (Third month)

Subgroup	Anti-CGRP Events	Anti-CGRP Total	Placebo Events	Placebo Total	Weight	Risk Ratio M-H, Random, 95% CI
**2.3.1 Response rate 50%, week 12 (Eptinezumab)**						
NCT01772524	56	73	52	78	16.8%	1.15 [0.94, 1.41]
**Subtotal (95% CI)**		**73**		**78**	**16.8%**	**1.15 [0.94, 1.41]**
Total events	56		52			
Heterogeneity: Not applicable						
Test for overall effect: Z = 1.37 (P = 0.17)						
**2.3.2 Response rate 50%, week 12 (Erenumab)**						
NCT02066415	152	375	66	281	15.1%	1.73 [1.35, 2.20]
NCT02456740	282	630	83	316	16.7%	1.70 [1.39, 2.09]
NCT02483585	112	282	85	288	15.7%	1.35 [1.07, 1.69]
NCT03096834	36	119	17	124	7.0%	2.21 [1.31, 3.71]
**Subtotal (95% CI)**		**1406**		**1009**	**54.5%**	**1.63 [1.39, 1.91]**
Total events	582		251			
Heterogeneity: Tau² = 0.01; Chi² = 4.40, df = 3 (P = 0.22); I² = 32%						
Test for overall effect: Z = 6.03 (P < 0.00001)						
**2.3.3 Response rate 50%, week 12 (Frenamezumab)**						
NCT02021773	93	172	28	89	11.6%	1.72 [1.23, 2.41]
**Subtotal (95% CI)**		**172**		**89**	**11.6%**	**1.72 [1.23, 2.41]**
Total events	93		28			
Heterogeneity: Not applicable						
Test for overall effect: Z = 3.16 (P = 0.002)						
**2.3.4 Response rate 50%, week 12 (Galcanezumab)**						
NCT02163993	47	62	78	126	17.1%	1.22 [1.01, 1.49]
**Subtotal (95% CI)**		**62**		**126**	**17.1%**	**1.22 [1.01, 1.49]**
Total events	47		78			
Heterogeneity: Not applicable						
Test for overall effect: Z = 2.02 (P = 0.04)						
**Total (95% CI)**		**1713**		**1302**	**100.0%**	**1.48 [1.26, 1.75]**
Total events	778		409			
Heterogeneity: Tau² = 0.03; Chi² = 18.40, df = 6 (P = 0.005); I² = 67%						
Test for overall effect: Z = 4.68 (P < 0.00001)						
Test for subgroup differences: Chi² = 10.34, df = 3 (P = 0.02), I² = 71.0%						

Risk Ratio
M-H, Random, 95% CI

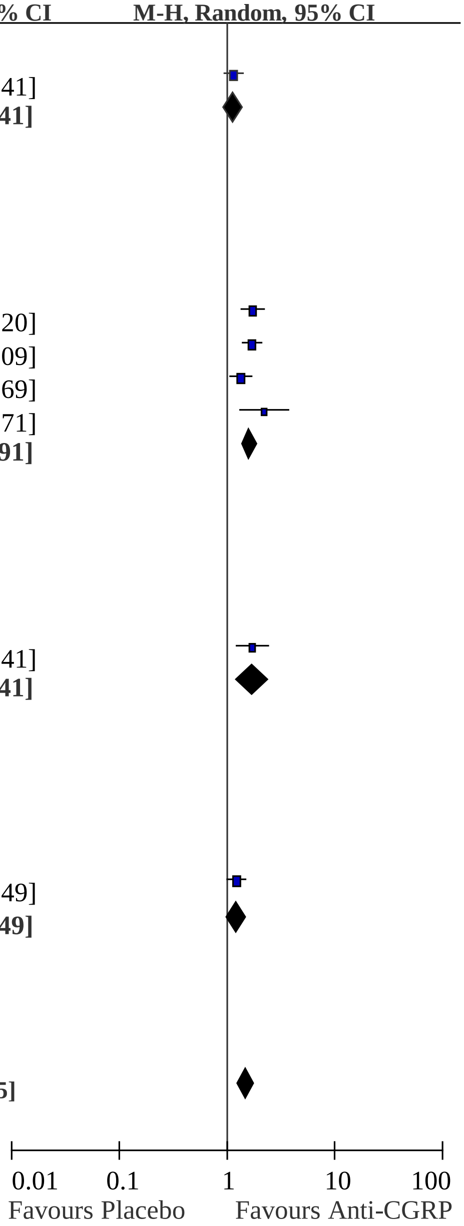

Figure S7 to S9
Small study effect of the cumulative response rates

Figure S7

Small study effect test for Cumulative response rate (50%)

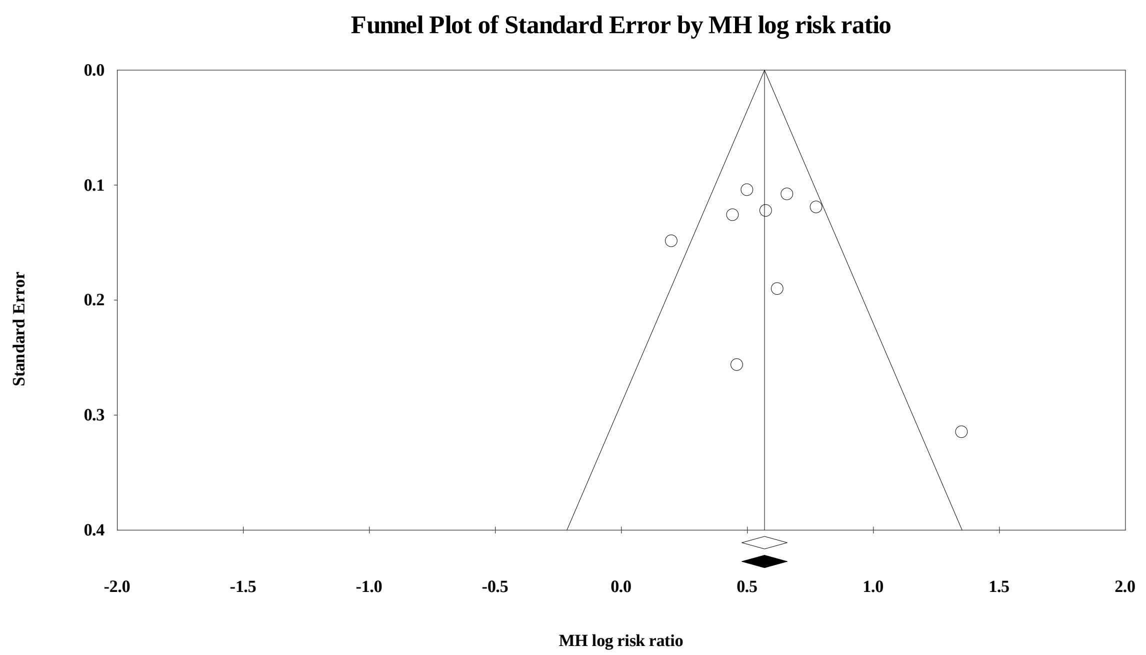

Egger's regression intercept

Intercept	1.08945
Standard error	1.70765
95% lower limit (2-tailed)	-2.94849
95% upper limit (2-tailed)	5.12739
t-value	0.63798
df	7.00000
P-value (1-tailed)	0.27189
P-value (2-tailed)	0.54378

Figure S8

Small study effect test for Cumulative response rate (75%)

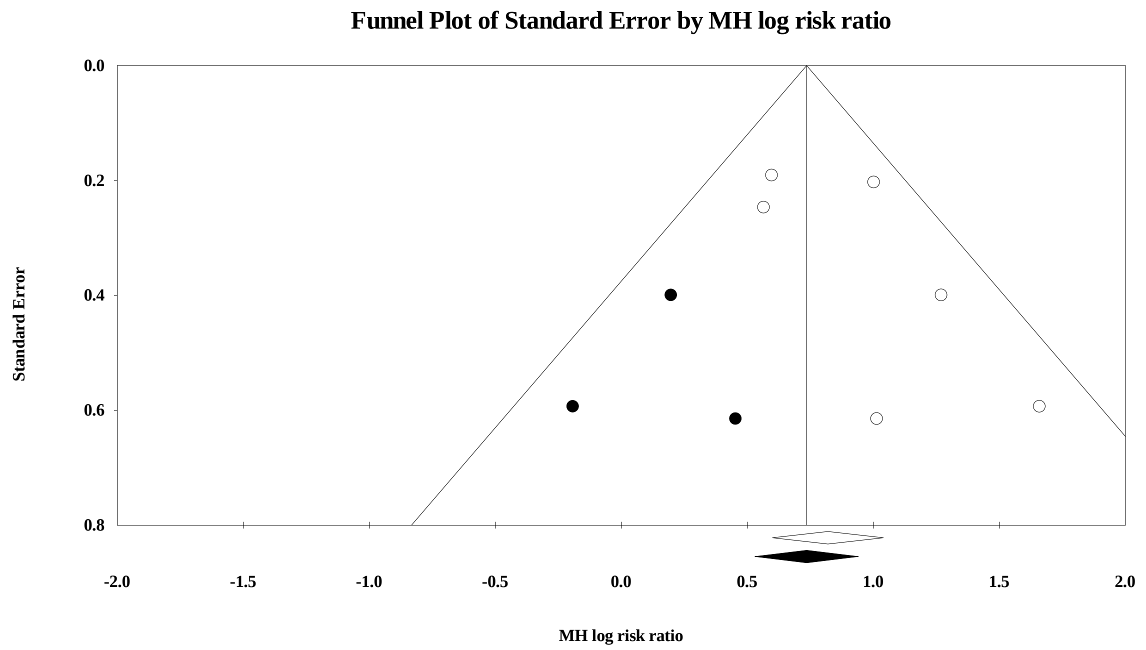

Egger's regression intercept

Intercept	1.63319
Standard error	1.01045
95% lower limit (2-tailed)	-1.17228
95% upper limit (2-tailed)	4.43866
t-value	1.61630
df	4.00000
P-value (1-tailed)	0.09067
P-value (2-tailed)	0.18134

Figure S9

Small study effect test for Cumulative response rate (100%)

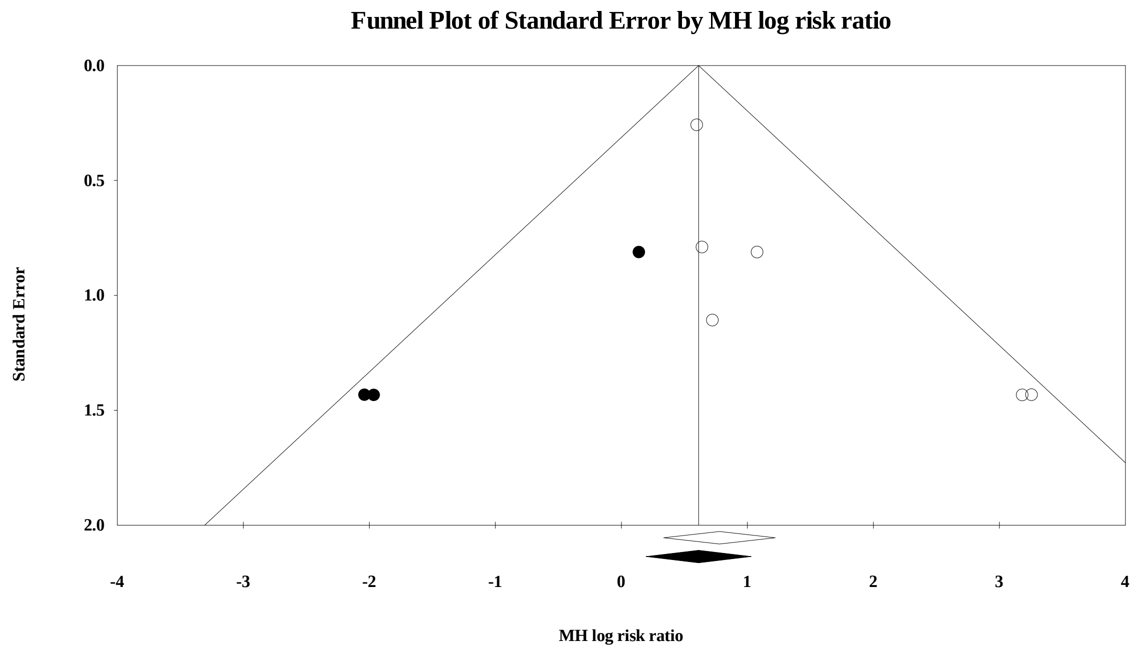

Egger's regression intercept

Intercept	1.31031
Standard error	0.54602
95% lower limit (2-tailed)	-0.20570
95% upper limit (2-tailed)	2.82632
t-value	2.39973
df	4.00000
P-value (1-tailed)	0.03719
P-value (2-tailed)	0.07438

Figure S10 to S12
Further analysis of cumulative response rates

Figure S10

Subset analysis for cumulative response rate (50%)

Subgroup	Anti-CGRP Events	Anti-CGRP Total	Placebo Events	Placebo Total	Weight	Risk Ratio M-H, Random, 95% CI
**2.4.1 Response rate 50%, from baseline to week 12 (Eptinezumab)**						
NCT01772524	41	67	25	76	8.7%	1.86 [1.28, 2.70]
**Subtotal (95% CI)**		**67**		**76**	**8.7%**	**1.86 [1.28, 2.70]**
Total events	41		25			
Heterogeneity: Not applicable						
Test for overall effect: Z = 3.26 (P = 0.001)						
**2.4.2 Response rate 50%, from baseline to week 12 (Erenumab)**						
NCT01952574	108	296	43	144	11.4%	1.22 [0.91, 1.64]
NCT02066415	225	566	88	428	14.6%	1.93 [1.56, 2.39]
**Subtotal (95% CI)**		**862**		**572**	**26.0%**	**1.55 [0.99, 2.44]**
Total events	333		131			
Heterogeneity: $Tau^2 = 0.09$; $Chi^2 = 6.24$, df = 1 (P = 0.01); $I^2 = 84\%$						
Test for overall effect: Z = 1.92 (P = 0.05)						
**2.4.3 Response rate 50%, from baseline to week 12 (Frenamezumab)**						
NCT02021773	49	172	16	89	5.9%	1.58 [0.96, 2.62]
NCT02025556	71	191	10	104	4.3%	3.87 [2.09, 7.17]
NCT02621931	294	750	67	371	13.7%	2.17 [1.72, 2.74]
NCT02629861	265	575	81	290	15.0%	1.65 [1.34, 2.03]
**Subtotal (95% CI)**		**1688**		**854**	**38.8%**	**2.02 [1.52, 2.70]**
Total events	679		174			
Heterogeneity: $Tau^2 = 0.05$; $Chi^2 = 8.83$, df = 3 (P = 0.03); $I^2 = 66\%$						
Test for overall effect: Z = 4.81 (P < 0.00001)						
**2.4.4 Response rate 50%, initial to week 12 (Galcanezumab)**						
NCT01625988	69	98	47	104	13.1%	1.56 [1.22, 2.00]
NCT02614261	150	547	83	538	13.4%	1.78 [1.40, 2.26]
**Subtotal (95% CI)**		**645**		**642**	**26.5%**	**1.67 [1.40, 1.98]**
Total events	219		130			
Heterogeneity: $Tau^2 = 0.00$; $Chi^2 = 0.61$, df = 1 (P = 0.43); $I^2 = 0\%$						
Test for overall effect: Z = 5.81 (P < 0.00001)						
**Total (95% CI)**		**3262**		**2144**	**100.0%**	**1.78 [1.54, 2.05]**
Total events	1272		460			
Heterogeneity: $Tau^2 = 0.02$; $Chi^2 = 18.00$, df = 8 (P = 0.02); $I^2 = 56\%$						
Test for overall effect: Z = 7.88 (P < 0.00001)						
Test for subgroup differences: $Chi^2 = 1.65$, df = 3 (P = 0.65), $I^2 = 0\%$						

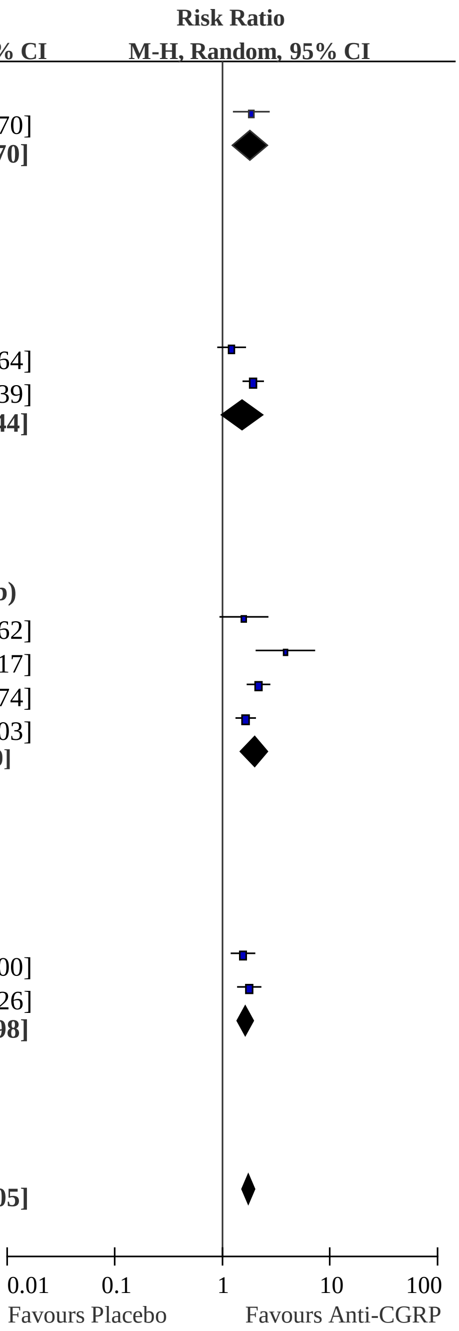

Figure S11

Subset analysis for cumulative response rate (75%)

Subgroup	Anti-CGRP Events	Anti-CGRP Total	Placebo Events	Placebo Total	Weight	Risk Ratio M-H, Random, 95% CI
**2.5.1 Response rate 75%, from baseline to week 12 (Eptinezumab)**						
NCT01772524	22	67	7	76	10.5%	3.57 [1.63, 7.81]
**Subtotal (95% CI)**		**67**		**76**	**10.5%**	**3.57 [1.63, 7.81]**
Total events	22		7			
Heterogeneity: Not applicable						
Test for overall effect: Z = 3.18 (P = 0.001)						
**2.5.2 Response rate 75%, from baseline to week 12 (Erenumab)**						
NCT02066415	101	566	28	428	27.8%	2.73 [1.83, 4.07]
**Subtotal (95% CI)**		**566**		**428**	**27.8%**	**2.73 [1.83, 4.07]**
Total events	101		28			
Heterogeneity: Not applicable						
Test for overall effect: Z = 4.93 (P < 0.00001)						
**2.5.3 Response rate 75%, from baseline to week 12 (Frenamezumab)**						
NCT02021773	16	172	3	89	4.9%	2.76 [0.83, 9.22]
NCT02025556	29	191	3	104	5.2%	5.26 [1.64, 16.86]
**Subtotal (95% CI)**		**363**		**193**	**10.1%**	**3.85 [1.67, 8.91]**
Total events	45		6			
Heterogeneity: $Tau^2 = 0.00$; $Chi^2 = 0.58$, df = 1 (P = 0.45); $I^2 = 0\%$						
Test for overall effect: Z = 3.16 (P = 0.002)						
**2.5.4 Response rate 75%, from baseline to week 12 (Galcanezumab)**						
NCT01625988	48	98	28	104	29.8%	1.82 [1.25, 2.65]
NCT02614261	43	547	24	538	21.8%	1.76 [1.08, 2.86]
**Subtotal (95% CI)**		**645**		**642**	**51.6%**	**1.80 [1.34, 2.42]**
Total events	91		52			
Heterogeneity: $Tau^2 = 0.00$; $Chi^2 = 0.01$, df = 1 (P = 0.92); $I^2 = 0\%$						
Test for overall effect: Z = 3.87 (P = 0.0001)						
**Total (95% CI)**		**1641**		**1339**	**100.0%**	**2.34 [1.77, 3.09]**
Total events	259		93			
Heterogeneity: $Tau^2 = 0.03$; $Chi^2 = 6.80$, df = 5 (P = 0.24); $I^2 = 26\%$						
Test for overall effect: Z = 6.01 (P < 0.00001)						
Test for subgroup differences: $Chi^2 = 5.99$, df = 3 (P = 0.11), $I^2 = 49.9\%$						

Risk Ratio
M-H, Random, 95% CI

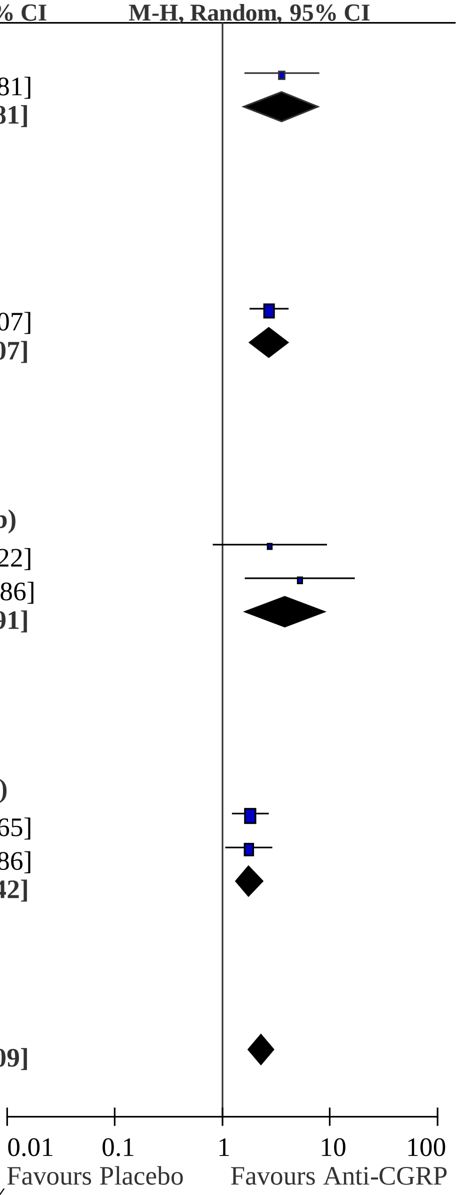

Figure S12

Subset analysis for cumulative response rate (100%)

Subgroup	Anti-CGRP Events	Anti-CGRP Total	Placebo Events	Placebo Total	Weight	Risk Ratio M-H, Random, 95% CI
**2.6.1 Response rate 100%, from baseline to week 12 (Eptinezumab)**						
NCT01772524	11	67	0	76	7.1%	26.04 [1.56, 433.71]
**Subtotal (95% CI)**		**67**		**76**	**7.1%**	**26.04 [1.56, 433.71]**
Total events	11		0			
Heterogeneity: Not applicable						
Test for overall effect: Z = 2.27 (P = 0.02)						
**2.6.2 Response rate 100%, from baseline to week 12 (Erenumab)**						
NCT02456740	11	76	0	80	7.1%	24.19 [1.45, 403.56]
**Subtotal (95% CI)**		**76**		**80**	**7.1%**	**24.19 [1.45, 403.56]**
Total events	11		0			
Heterogeneity: Not applicable						
Test for overall effect: Z = 2.22 (P = 0.03)						
**2.6.3 Response rate 100%, from baseline to week 12 (Frenamezumab)**						
NCT02021773	4	172	1	89	10.8%	2.07 [0.23, 18.24]
NCT02025556	7	191	2	104	17.4%	1.91 [0.40, 9.01]
**Subtotal (95% CI)**		**363**		**193**	**28.2%**	**1.96 [0.55, 6.94]**
Total events	11		3			
Heterogeneity: Tau² = 0.00; Chi² = 0.00, df = 1 (P = 0.95); I² = 0%						
Test for overall effect: Z = 1.04 (P = 0.30)						
**2.6.4 Response rate 100%, from baseline to week 12 (Galcanezumab)**						
NCT01625988	31	98	18	104	40.9%	1.83 [1.10, 3.05]
NCT02614261	6	547	2	538	16.8%	2.95 [0.60, 14.55]
**Subtotal (95% CI)**		**645**		**642**	**57.7%**	**1.91 [1.17, 3.11]**
Total events	37		20			
Heterogeneity: Tau² = 0.00; Chi² = 0.32, df = 1 (P = 0.57); I² = 0%						
Test for overall effect: Z = 2.61 (P = 0.009)						
**Total (95% CI)**		**1151**		**991**	**100.0%**	**2.93 [1.30, 6.57]**
Total events	70		23			
Heterogeneity: Tau² = 0.35; Chi² = 7.86, df = 5 (P = 0.16); I² = 36%						
Test for overall effect: Z = 2.61 (P = 0.009)						
Test for subgroup differences: Chi² = 6.11, df = 3 (P = 0.11), I² = 50.9%						

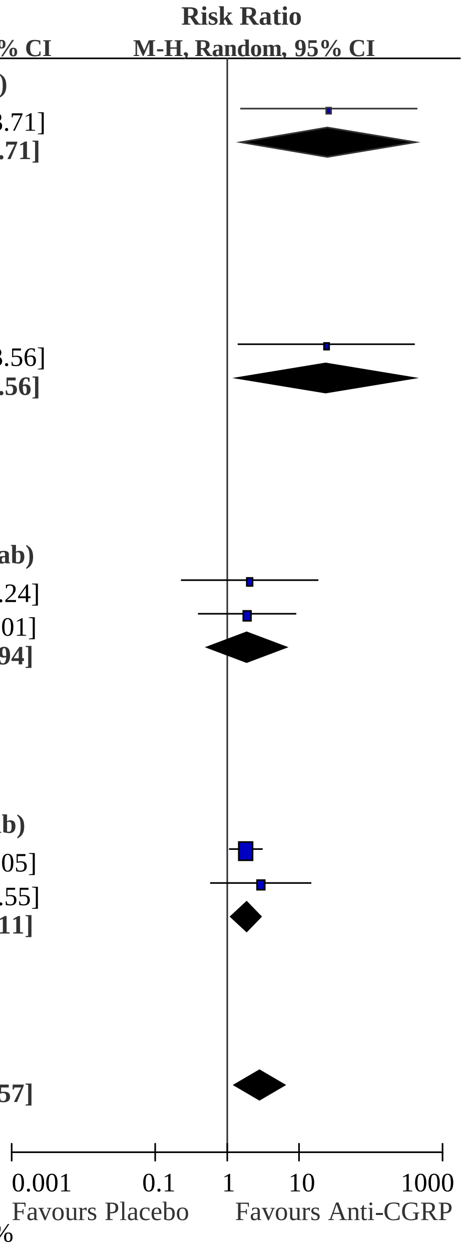

Supplement: Supplementary file 1 [file ijms-20-03527-s001.pdf]
